# Dissociative Electron Transfer to Diphenyl-Substituted Bicyclic Endoperoxides: The Effect of Molecular Structure on the Reactivity of Distonic Radical Anions and Determination of Thermochemical Parameters

**DOI:** 10.3390/molecules190811999

**Published:** 2014-08-11

**Authors:** David C. Magri, Mark S. Workentin

**Affiliations:** 1 Department of Chemistry, University of Malta, Msida, MSD 2080, Malta; 2 Department of Chemistry, The University of Western Ontario, London, ON N6A 5B7, Canada

**Keywords:** dissociative electron transfer, distonic radical ion, endoperoxide, convolution analysis, electrode interface, cyclic voltammetry

## Abstract

The heterogeneous electron transfer reduction of the bicyclic endoperoxide 1,4-diphenyl-2,3-dioxabicyclo[2.2.1]hept-5-ene (**4**) was investigated in *N*,*N*-dimethylformamide at a glassy carbon electrode. The endoperoxide reacts by a concerted dissociative ET mechanism resulting in reduction of the O-O bond with an observed peak potential of −1.4 V at 0.2 V s^−1^. The major product (90% yield) resulting from the heterogeneous bulk electrolysis of **4** at −1.4 V with a rotating disk glassy carbon electrode is 1,4-diphenyl-cyclopent-2-ene-*cis*-1,3-diol with a consumption of 1.73 electrons per mole. In contrast, 1,4-diphenyl-2,3-dioxabicyclo[2.2.2]oct-5-ene (**1**), undergoes a two-electron reduction mechanism in quantitative yield. This difference in product yield between **1** and **4** is suggestive of a radical-anion mechanism, as observed with 1,4-diphenyl-2,3-dioxabicyclo-[2.2.2] octane (**2**) and 1,4-diphenyl-2,3-dioxabicyclo[2.2.1]heptane (**3**). Convolution potential sweep voltammetry is used to determine unknown thermochemical parameters of **4**, including the O-O bond dissociation energy and the standard reduction potential and a comparison is made to the previously studied bicyclic endoperoxides **1**–**3** with respect to the effect of molecular structure on the reactivity of distonic radical anions.

## 1. Introduction

Radical ions are an important class of reactive intermediate possessing dual reactivity with properties of both a radical and an ion [1]. They are involved in many crucial biological and synthetic organic chemistry processes and materials science applications [2]. Typically, they result from the transfer of a single electron to a neutral molecule to yield an intermediate possessing both a charge and radical character, or from the ionization of zwitterions or diradicals [3]. Both theoretical and experimental work has converged to provide convincing evidence that both charge and spin are important factors in the reactivity of radical ions [4,5,6,7]. Coote and her team have recently provided convincing evidence that in the gas phase a sufficiently stabilized localized radical linked by an aliphatic carbon spacer can be stabilized by a negative charge [8,9].

**Figure 1:**
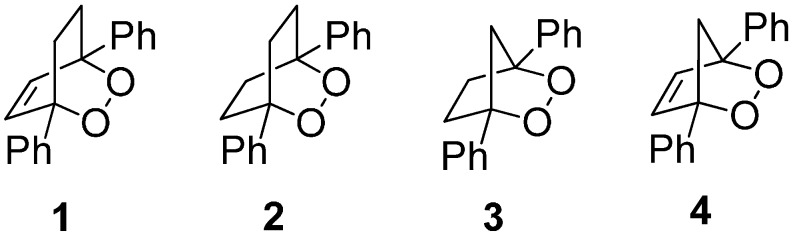
The molecular structures of bicyclic endoperoxides **1**–**4**.

The heterogeneous reduction of aliphatic peroxides [10,11,12,13] and peresters [14,15,16,17] in aprotic solvents, such as acetonitrile and *N*,*N*-dimethylformamide (DMF), has been shown to occur by a dissociative electron transfer (ET) mechanism. Similarly, the heterogeneous reduction of endoperoxides has also been consistent with a dissociative ET mechanism [18,19,20,21,22,23,24,25] and including photo-initiated examples [26,27]. The first step of the mechanism involves O-O bond cleavage by ET to form a distonic radical-anion, a reactive intermediate with a spatially separated radical and anion (Equation (1)). Subsequently, the major competitive pathway is reduction of the distonic radical anion at the electrode, or in solution by an electrochemically-generated radical-anion donor, to the dialkoxide, which is protonated to yield the *cis*-diol (Equation (2)). The reduction of the distonic radical is highly favourable under electrochemical conditions as the reduction potential is much more positive than the initial reduction of the endoperoxide.

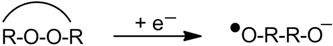
(1)

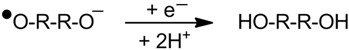
(2)
We have shown that diphenyl-substituted endoperoxides that form a distonic radical anion upon O-O cleavage may undergo a fragmentation reaction in competition with the second heterogeneous ET [18,19,20,21,22]. We have previously reported the ET-initiated reduction of the bicyclic endoperoxides 1,4-diphenyl-2,3-dioxabicyclo[2.2.2]oct-5-ene (**1**, Figure 1) [18], 1,4-diphenyl-2,3-dioxabicyclo[2.2.2] octane (**2**, Figure 1) [18], and 1,4-diphenyl-2,3-dioxabicyclo[2.2.1]heptane (**3**, Figure 1) [19] using heterogeneous electrochemical techniques. In these studies, we evaluated previously unknown kinetic and thermochemical data by application of Savéant’s theory of dissociative ET [28,29,30,31,32]. With the dialkyl bicyclic endoperoxides, ascaridole and dihydroascaridole, we observed that reduction occurred by a two-electron reductive mechanism yielding the *cis*-diol in quantitative yield [22]. The same two-electron reductive mechanism was observed with the diphenyl-substituted endoperoxide (**1**). However, in the case of **2** and **3** the distonic radical anion was observed to undergo a competitive β-scission fragmentation resulting in a propagating radical-anion chain mechanism initiated by dissociative ET reduction of a bicyclic endoperoxide [18].

In this study we report the heterogeneous reduction of 1,4-diphenyl-2,3-dioxabicyclo[2.2.1]hept-5-ene (**4**, Figure 1) at a glassy carbon electrode. The approach is advantageous as it is relatively easy to determine the activation-driving force relationship from the obtained current-potential response. The endoperoxide was studied using a number of electrochemical techniques including cyclic voltammetry, convolution potential sweep voltammetry and preparative electrolysis in order to determine unknown thermochemical parameters, including the O-O bond dissociation energy and the standard reduction potential and to gain insight into the reactivity of the distonic radical anion resulting from cleavage of the O-O bond.

## 2. Results and Discussion

### 2.1. Cyclic Voltammetry and Constant Potential Electrolyses

**Figure 2:**
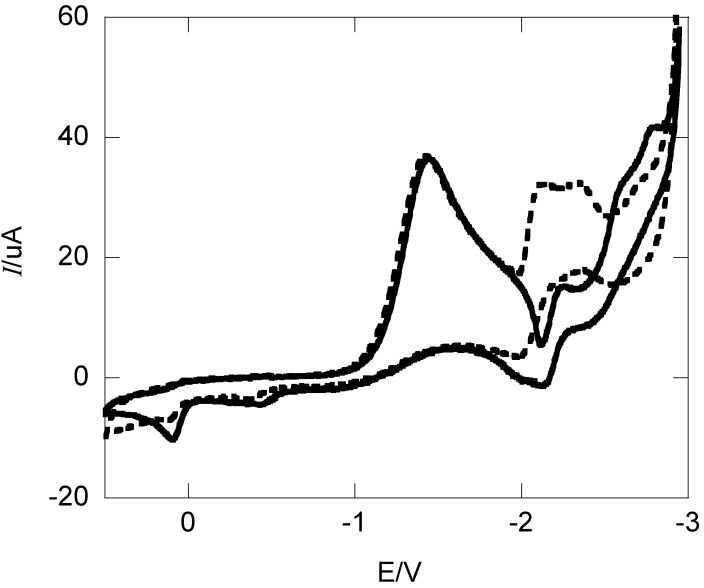
Cyclic voltammograms of 1.84 mM of **4** in a solution of DMF containing 0.10 M TEAP in the absence (solid line) and presence (dashed line) of 10 equivalents of 2,2,2-trifluoroethanol at 0.1 V s^−1^.

The electrochemical reduction of 1,4-diphenyl-2,3-dioxabicyclo[2.2.1]hept-5-ene (**4**) was studied by cyclic voltammetry using a glassy carbon electrode in DMF containing 0.10 M tetraethylammonium perchlorate (TEAP). The voltammogram of **4**, as shown in Figure 2, is characterized by an electrochemically irreversible cathodic peak with a peak potential, *E*_p_ of −1.42 V versus SCE at 0.1 V s^−1^. On increasing the scan rate the *E*_p_ shifts to −1.53 and −1.63 V at 1.0 and 10 V s^−1^ respectively. With increasing scan rate *v* the peak width at half height (Δ*E*_p/2_) broadens from 178 to 214 mV with a negative shift of 132 mV per log decade. The transfer coefficient α, determined from the peak widths using α = 1.857RT/(FΔ*E*_p_/2) are found to decrease with *v* from 0.268 to 0.223 [31]. Using the scan rate dependence of the peak potential and α = 1.15RT/[*d*Ep/(*d*log *v*)], α is 0.26 indicating the transition state closely resembles the initial reaction state. These characteristics of the voltammogram are consistent with a concerted dissociative mechanism ET mechanism. On scanning back after the forward reduction at a switching potential of −1.9 V, poorly defined anodic peaks are observed between 0.1 and −0.5 V due to oxidation of the dialkoxide anion. A summary of voltammetry data is included in Table 1 along with previous data for endoperoxides **1**–**3** for comparison. For **4** the *E*_p_ is rather similar to the other endoperoxides, whereas the Δ*E*_p/2_ is somewhat broader. The α values are low characteristic of an outright concerted dissociative ET mechanism.

When the switching potential is extended past the end of the initial wave, which is normally dictated exclusively by diffusion, a sharp oxidative dip is observed at −2 V. This feature of the voltammograms is most noticeable at slower scan rates yet remains visible up to 10 V s^−1^. Following the dip at more negative scanned potentials there are other cathodic and anodic peaks attributed to products resulting from the reduction of the **4** following dissociative reduction of the O-O bond. However, the addition of excess weak acid results in an increase in peak current −2 V even at a scan rate of 0.1 V s^−1^ (See Figure 2). These peaks are due to electroactive products resulting from the initial reduction of the O-O bond.

**Table 1:** Cyclic voltammetry data for the endoperoxides **1**–**4** in 0.10 M TEAP/DMF at 25 °C measured at a glassy carbon electrode.

Experimental CV Data	1	2	3	4
*E*_p_/V @ 0.1 V s^−1^	−1.27	−1.45	−1.34	−1.42
Δ*E*_p/2_/mV @ 0.1 V s^−1^	136	142	116	178
α @ 0.1 V s^−1^	0.351	0.336	0.411	0.268
*(dE*_p_/*d*log ν)/mV^−1^ ^a^	−118	−105	−132	−115
α = 1.15RT/F(*dE*_p_/*d*log ν) ^a^	0.25	0.28	0.23	0.26
*n* @ *E*_p_ (no acid) ^b^	1.98	1.94	1.97	1.73
*n* @ *E*_p_ (acid) ^b^	2.08	1.96	1.92	1.74
*n* @ *E* (ca. 200 mV past dip) ^b^	1.96	1.3	0.8	0.8

^a^ Scan rate range of 0.1 to 10 V s^−1^. ^b^ Electrolyses performed with a glassy carbon rotating disc electrode.

At potentials between −1.4 to −2.0 V, between the dissociative wave and the dip, **4** consumes 1.7 F mol^−1^ of charge independent of acid present. A redox couple is observed at −2.16 V after complete disappearance of the dissociative wave, followed by other cathodic peaks at more negative potentials. The major product recovered from the electrolysis mixture in 90% yield is 1,4-diphenylcyclopent-2-ene-*cis*-1,3-diol. Electrolyses conducted at −2.2 V result in the consumption of only 0.8 F mol^−1^ of charge, and the appearance of electroactive products at more negative potentials at the expense of the dissociative wave.

### 2.2. Heterogeneous Kinetics and Thermochemical Parameters

**Figure 3:**
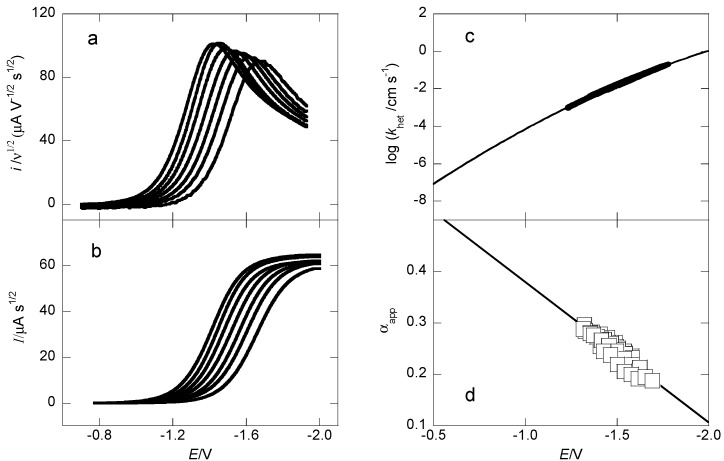
(**a**) Background-subtracted linear sweep voltammograms of 2.0 mM of **4** in DMF containing 0.10 mol L^−1^ TEAP (from left to right: 0.1, 0.2, 0.4, 1.0, 2.0, 4.0, 10 V s^−1^); (**b**) the convolution curves as a function of scan rate; (**c**) overlapping potential dependence of the log *k*_het_; (**d**) the potential dependence of α_app_ at 10 scan rates between 1.0 and 20 V s^−1^.

The heterogeneous ET kinetics and thermochemical parameters for reduction of the O-O bond were evaluated by convolution potential sweep voltammetry [33,34,35]. Scan rates were varied from 0.10 to 10 V s^−1^ as observed in Figure 3a. The faster scan rates were the most reliable because of non-Cottrell behaviour at the slower scan rates. A total of 20 background-subtracted cyclic voltammograms were recorded and transformed into sigmoidal-shaped *I*-*E* curves by use of the convolution integral (Equation (3)) and the experimental current *i* [33,34,35].


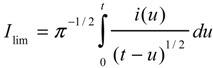
(3)

The limiting current, *I*_lim_, at the plateau of the sigmoidal-shaped *i*-*E* curves is diffusion-controlled and defined as *I*_lim_ = nFAD^1/2^C* where *n* is the overall electron consumption, *A* is the electrode area, *D* is the diffusion coefficient, and C* is the substrate concentration. For an irreversible electrode process *I*_lim_ and *i* are related to *k*^1^_het_ by:

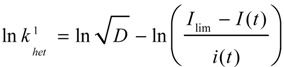
(4)

Derivatisation of the ln *k*^1^_het_ allows the evaluation of the apparent transfer coefficient, α_app_, uncorrected for the double layer:

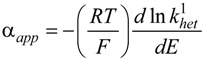
(5)

The error between the limiting plateau curves was found to be within 5% (Figure 3b). From the limiting currents, *I*_lim_, and the known area of the glassy carbon electrode, the diffusion coefficient in the presence of 0.1 M TEAP was determined to be 6.0 × 10^−6^ cm^2^ s^−1^. From the potential dependence of α_app_ for a concerted dissociative mechanism, *E*°_diss_ was determined to be −0.56 V from extrapolation of an α_app_-E plot corresponding to α_app_ = 0.5 (Figure 3d). The slope of the linear regression provided an intrinsic barrier of 10.6 kcal mol^−1^ (Figure 3d). The log *k*°_het_ was evaluated to be −6.7 (Figure 3c), which is consistent with a totally irreversible rate-determining electrode reaction characteristic of concerted dissociative ET

In Table 2 the parameters from the convolution analysis are summarized along with previous results for endoperoxides **1**–**4**. As determined from the α_app_-E plots, the evaluated *E*°_diss_ and ∆*G*_o_^≠^ for **1**–**4** are consistent with average values of −0.56 V and 10.7 kcal mol^−1^, respectively. The *E*°_diss_ is substantially more positive than the *E*_p_ by over 0.8 V due to a large overpotential attributed to the large ∆*G*_o_^≠^ that must be overcome to stretch and break the O-O bond. The accuracy of the evaluated thermochemical parameters in acidic media were adequately reproduced by digital simulation of the dissociative waves using the data in Table 1 and Table 2 for a two-electron concerted dissociative mechanism.

**Table 2:** Data acquired by convolution analysis of diphenyl-substituted bicyclic endoperoxides **1**–**4** in DMF containing 0.10 mol L^−1^ TEAP at 25 °C at a glassy carbon electrode.

Thermodynamic Data	1	2	3 ^g^	4
*D*/cm^2^ s^−1^ ^a^	7.7 ° 10^−6^	7.4 ° 10^−6^	6.5 ° 10^−6^	6.0 ° 10^−6^
*E*°_diss_/V ^b^	−0.61	−0.51	−0.54	−0.56
log (*k*°_het_/cm s^−1^) ^c^	−6.1	−6.5	−6.8	−6.7
Δ*G*_o_^≠^/kcal mol^−1^ ^d^	9.8	10.4	11.8	10.6
λ_het_/kcal mol^−1^ ^e^	20	20	18	19
BDE/kcal mol^−1^ ^f^	19	22	20 ^h^	20 ^i^

^a^ Determined from the convoluted limiting current and the known area of the electrode. The area of the electrode was calculated using a diffusion coefficients for ferrocene of 1.13 × 10^−5^ cm^2^ s^−1^ in DMF [11]. ^b^ Estimated error is ±0.10 V and uncorrected for the double layer. ^c^ Determined by extrapolation of the heterogeneous kinetics from a second order polynomial fit. Estimated error of ±0.5 from digital simulation. ^d^ Δ*G*_o_^≠^ determined from the slope of α_app_ *vs.* *E* plots and Δ*G*_o_^≠^ = F/[8(dα/dE)]. ^e^ Based on empirical relationship λ_het_ = 55.7/*r*_AB_. The radii *r*_AB_ was calculated from the Stokes-Einstein equation and diffusion coefficients to be 3.6, 3.7, 4.6 and 4.2 Å for **1**–**4**, respectively. The effective radius were calculated using *r*_eff_ = *r*_B_(2*r*_AB_ − *r*_B_)/*r*_AB_ to give 2.8, 2.8, 3.0 and 2.9 Å for **1**–**4**, respectively. ^f^ BDE = 4Δ*G*_o_^≠^ − λ_het_. ^g^ Based on scan rates between 1.0 and 20 V s^−1^ while all others between 0.1 and 20 V s^−1^. ^h^ Corrected for inner reorganization energy of up to 9 kcal mol^−1^ [19]. ^i^ Corrected for inner reorganization energy of 4 kcal mol^−1^.

**Scheme 1:**
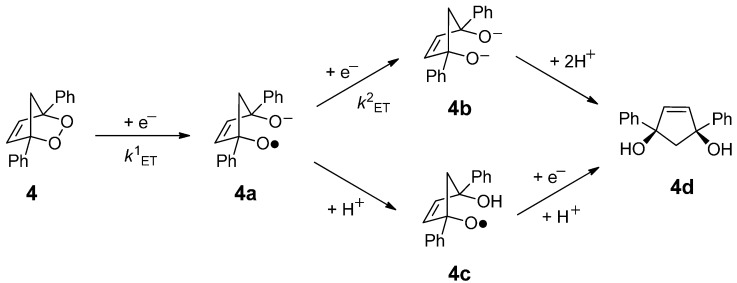
The predominant mechanistic pathways to 1,3-diphenylcyclopentene-1,3-*cis*-diol (**4d**) on reduction of the bicyclic endoperoxide **4** with a rotating disc glassy carbon electrode.

Scheme 1 depicts the heterogeneous ET reduction mechanism of **4** to yield the *cis*-diol. A near quantitative yield of 1,3-diphenylcyclopentene-*cis*-1,3-diol (**4d**) was obtained in 90% yield. The reduction is initiated by DET to the σ* orbital mainly localized on the O-O bond, resulting in homogeneous cleavage and the formation of the distonic radical-anion **4a** with a negative charge on one oxygen atom and an unpaired electron on the other. The distonic radical anion can then subsequently react in one of two ways to yield the *cis*-diol **4d**. Either **4a** generated at the interface of the electrode, could be reduced to the dialkoxide **4b** before diffusing away from the electrode, followed by protonation in the reaction solution or upon work-up to yield **4d**. Alternatively, notably in the presence of a weak acid, **4a** could be protonated resulting in the alkoxyl radical **4c**, which could be further reduced by the electrode and subsequently protonated to yield **4d**. At a potential of −1.5 V, the reduction of both **4a** and **4c** are predicted to be thermodynamically favorable by at least 25 kcal mol^−1^. 

A summary of the thermochemical parameters determined by convolution potential sweep voltammetry are shown in Table 2. The *E*°_diss_ is related to the O-O bond dissociation energy (BDE) by the equation *E*°_diss_ = *E*°_•ORRO^−^_ − BDFE/*F* derived from a thermochemical cycle where *E*°_•ORRO^−^_ is the standard reduction potential of the distonic radical anion, *F* is Faraday’s constant and the BDFE is the bond dissociation free energy, which is the BDE corrected for entropy (BDFE = BDE − *T*Δ*S*). The *E*°_•ORRO^−^_ was approximated from the standard potential of the cumyl alkoxyl radical equal to −0.12 V [11]. Using the above equation, the BDFE of **4** is 10 kcal mol^−1^ consistent with previous calculations for the related diphenyl-substituted endoperoxide series [18,19]. The BDE was determined using Savéant’s concerted dissociative ET model [31] from the experimental Δ*G*_o_^≠^ and the expression Δ*G*_o_^≠^ = (λ_het_ + BDE)/4, where λ_het_ is the solvent reorganization energy. The empirical relation λ_het_ = 55.7/*r*_AB_, was used where *r*_AB_ is the molecular radius, given in angstroms, as determined from the Stokes-Einstein equation and the experimentally determined diffusion coefficient. An effective radius approach was used in the calculations (see Table 2 footnotes). The *r*_eff_ was estimated to be 2.9 Å and leading to λ_het_ equal to 19 kcal mol^−1^ and a BDE of 23 kcal mol^−1^. The slightly higher BDE compared to **1** and **3** is suggestive of a contribution from the relief of ring strain upon fragmentation of the O-O within the bicyclo[2.2.1]hept-5-ene structure. Endoperoxide **3** was estimated to have an inner reorganization energy of 9 kcal mol^−1^ [19]. Following the same approach, **4** has an inner reorganization energy of 4 kcal mol^−1^.

A final comparison of the kinetic and thermodynamic parameters of **4** with **1** and **2** and **3** is worthy of remarks (Table 2). The *k*°_het_ and *E*°_diss_ are identical within experimental error. The diffusion coefficients of **3** and **4** are slightly smaller than **1** and **2** due to the fact that the carbon skeleton is one carbon less and hence the molecular sphere radii are smaller. Using digital simulation, the extracted data from the convolution analysis was adequately reproduced using the experimental cyclic voltammograms of **4** taking into account the lower 1.7 electron stoichiometry by decreasing the initial concentration by 15%. This finding suggests the rapid fragmentation of the distonic radical anion may be producing a species not easily reduced at an electrode potential greater than −2.0 V.

## 3. Experimental Section

### 3.1. General Information

*N,N*-Dimethylformamide (DMF) was distilled over CaH_2_ under a nitrogen atmosphere at reduced pressure. Tetraethylammonium perchlorate (TEAP) was recrystallized three times from ethanol and stored in a vacuum oven. Other solvents and reagents not specified were used without purification. Melting points were recorded on an Electrothermal 9100 capillary melting point apparatus and were corrected. UV-visible spectra were recorded on a Varian Cary 100 Bio UV-visible spectrometer. Infrared spectra were recorded on a Bruker Vector 33 FT-IR spectrometer on NaCl plates or in a solution cell and are reported in cm^−1^. ^1^H and ^13^C-NMR spectra were recorded at 400.1 and 100.6 MHz, respectively, with CDCl_3_ as the solvent, on a Varian Mercury spectrometer and are reported in ppm. *versus* tetramethylsilane (δ_H_ = 0.00) for ^1^H-NMR and CDCl_3_ (δ_C_ = 77.00) for ^13^C-NMR. Mass spectrometry was performed on a MAT 8200 Finnigan high-resolution mass spectrometer by electron impact (EI) and by chemical ionisation (CI) with isobutane.

### 3.2. Synthesis of 1,4-Diphenyl-2,3-dioxabicyclo[2.2.1]hept-5-ene (**4**)

The endoperoxide was synthesised by photo-oxygenation of 1,4-diphenyl-1,3-cyclopentadiene as previously described [8]. The reaction was monitored by TLC following the disappearance under 365 nm UV light of the diene at *R*_f_ = 0.70 (1:1 hexanes/dichloromethane eluent). The product was purified by flash chromatography using 1:4 hexanes/dichloromethane and collected as the second eluant. The product was recrystallised from MeOH to yield white crystals. m.p. 105–107 °C; ν_max_ (NaCl)/cm^−1^: 3061, 3033, 2920, 2851, 1700, 1652, 1448, 1333, 1093, 1074, 1025, 887, 823, 749, 697; ^1^H-NMR (CDCl_3_, 400 MHz, ppm): δ_H_ 2.57 (AB, *J* = 8.6 Hz, 1H), 2.68 (AB, *J* = 8.6 Hz, 1H), 6.83 (s, 2H), 7.38–7.48 (m, 6H), 7.56–7.61 (m, 4H); ^13^C-NMR (CDCl_3_, 100 MHz, ppm): δ_C_ 61.12, 96.14, 126.88, 128.78, 129.12, 133.28, 137.89; MS *m/z* (% intensity): 250 (M^•+^, 1) 249 (3), 220 (4), 219 (18), 218 (100), 217 (15), 215 (5), 203 (8), 202 (6), 105 (9), 77 (13); Exact Mass (M^•+^−1): 249.0909 (calculated 249.0915).

### 3.3. Electrochemistry

Cyclic voltammetry was performed using either a Perkin-Elmer PAR 283 or 263A potentiostat interfaced to a personal computer equipped with PAR 270 electrochemistry software. The working electrode was a 3 mm diameter glassy carbon rod (GC-20, Tokai) sealed in glass tubing. The counter electrode was a 1 cm^2^ Pt plate. The reference electrode was a silver wire immersed in a glass tube with a sintered end containing 0.10 M TEAP in DMF. The reference electrode was calibrated against the ferrocene/ferrocenium redox couple after each experiment (0.475 V versus KCl saturated calomel electrode in DMF). Constant potential electrolyses were conducted with a 12 mm tipped glassy carbon rotating disk electrode (EDI101) with a CTV101 speed control unit from Radiometer Analytical [18].

### 3.4. Heterogeneous Electrolysis Products

1,4-Diphenyl-cyclopent-2-ene-*cis*-1,3-diol was recovered in 90% yield from the electrolysis of 1,4-diphenyl-2,3-dioxabicyclo[2.2.1]-hept-5-ene. ^1^H-NMR (CDCl_3_, ppm): δ_H_ 2.48 (AB, *J* = 14.2 Hz, 1H), 2.61 (AB, *J* = 14.2 Hz, 1H), 2.83 (s, br, 2H), 6.32 (s, 2H), 7.25–7.30 (m, 2H), 7.33–7.40 (m, 4H), 7.44–7.48 (m, 4H), alcohol peak verified by deuterium exchange; ^13^C-NMR (CDCl_3_, ppm): δ_C_ 58.95, 85.90, 124.94, 127.36, 128.42, 140.19, 144.80; MS *m/z* (% intensity): 253 (M^•+^+1,15), 252 (M^•+^,76), 235 (11), 234 (39), 233 (32), 205 (13), 133 (70), 120 (100), 105 (95), 91 (24), 47 (77); Exact Mass: 252.1142 (calculated 252.1150).

## 4. Conclusions

The bicyclic endoperoxide 1,4-diphenyl-2,3-dioxabicyclo[2.2.1]hept-5-ene (**4**) was studied using heterogeneous electrochemical techniques. It was found that reduction of the O-O bond occurs by a DET mechanism with thermodynamic and kinetic parameters consistent with the other diphenyl-substituted endoperoxides **1**–**3** [18,19]. In all cases, the electrode kinetics are slow and the O-O bond energy is low. This study suggests **4** reacts by a radical-anion chain mechanism initiated by the concerted DET reduction. The overall reactivity of **4** has many characteristics similar to **2** and **3** rather than a clear two-electron mechanism resulting in quantitative formation of *cis*-diol as with **1**. The elucidation of the complete reductive mechanism of **4** is subject to further investigation.
